# A Submersible Printed Sensor Based on a Monopole-Coupled Split Ring Resonator for Permittivity Characterization

**DOI:** 10.3390/s19081936

**Published:** 2019-04-25

**Authors:** Erick Reyes-Vera, G. Acevedo-Osorio, Mauricio Arias-Correa, David E. Senior

**Affiliations:** 1 Department of Electronic and Telecommunications Engineering, Instituto Tecnológico Metropolitano, Medellín 050012, Colombia; gabrielacevedo84945@correo.itm.edu.co; 2 Department of Mechatronics and Electromechanical Engineering, Instituto Tecnológico Metropolitano, Medellín 050034, Colombia; mauricioarias@itm.edu.co; 3 Department of Electrical and Electronic Engineering, Universidad Tecnologica de Bolivar, Cartagena 130001, Colombia; david.senior@ttm.com

**Keywords:** microwave sensor, split ring resonator, permittivity measurements, material characterization, metamaterial

## Abstract

This work presents a non-invasive, reusable and submersible permittivity sensor that uses a microwave technique for the dielectric characterization of liquid materials. The proposed device consists of a compact split ring resonator excited by two integrated monopole antennas. The sensing principle is based on the notch introduced by the resonators in the transmission coefficient, which is affected due to the introduction of the sensor in a new liquid material. Then, a frequency shift of the notch and the Q-factor of the proposed sensor are related with the changes in the surrounding medium. By means of a particular experimental procedure, commercial liquids are employed to obtain the calibration curve. Thus, a mathematical equation is obtained to extract the dielectric permittivity of liquid materials with unknown dielectric properties. A good match between simulated and experimental results is obtained, as well as a high Q-factor, compact size, good sensitivity and high repeatability for use in sensing applications. Sensors like the one here presented could lead to promising solutions for characterizing materials, particularly in determining material properties and quality in the food industry, bio-sensing and other applications.

## 1. Introduction

Permittivity is an important parameter used to describe the electromagnetic properties of dielectric materials [1,2]. In many areas of science and engineering, the ability to monitor and quantify the dielectric permittivity of materials using non-destructive methods with high sensitivity and precision is required. The measurement of permittivity is related to other characteristics of the material and can be used to determine changes in its density, concentration, composition, temperature, stress-strain tensor, among others [3,4,5,6,7,8]. For this reason, the measurement of this parameter is very important in many fields, some of which are agriculture [9,10], security [5], food quality [4], biology [11,12], among others. For example, the permittivity of vegetable oils is measured and evaluated according to the variation of the temperature and it has been established in [13], that an increasing temperature increases the permittivity of vegetable oil. Loss of meat moisture during the aging period is a critical issue for the meat industry, which is why in [4], a non-invasive microwave ring resonator sensor to evaluate the water holding capacity (WHC) of broiler meat was presented. Also, in the agricultural industry, measurement of permittivity is necessary to check the level of soil moisture, which has an important impact on food crops [9].

Several experimental methods have been employed to carry out dielectric permittivity property measurements for liquid, solid and gaseous materials. The main techniques include the free space technique, and the use of resonators, parallel plate capacitors, optical techniques and microwave circuit technology [2,14,15,16,17]. As a brief classification in the radio-frequency and microwave bands, methods can be split into non-resonance and resonance methods. The non-resonance methods include mainly the RF circuit method, the open ended coaxial probe and the free space method [2,6]. These are all non-destructive methods. Another non-resonance but destructive method is the transmission and reflection method, because a portion of the material under test (MUT) has to be situated inside the transmission line, so particular dimensions are required to ensure a correct fit [2,5]. Due to the low cost of development, real-time monitoring, easy integration and easy miniaturization, resonance methods have generated great interest in recent years. Resonance methods primarily include two types: resonant perturbation methods [7,18,19], and resonator methods [11,20,21,22,23]. In the resonant perturbation methods, a cavity resonator is filled with the sample under test (SUT), and the shift in the resonance frequency and the change in the quality factor are measured. With a precise sample preparation, this method becomes the most precise one, but is just applicable over a narrow band, and besides, this method is considered destructive if the material must be damaged to perform the test [5,14,19]. On the other side, the resonator methods are non-destructive since the SUT can be considered as part of the resonator and the permittivity can be deduced from the displacement of the relative frequency of resonance, which has a relatively high precision and sensitivity [2,21,24,25,26,27].

In the resonator methods, split ring resonators (SRR) have become common devices to obtain permittivity measurements. An SRR is an small electrical resonator that can be considered a metamaterial particle with simultaneous negative permeability and permittivity [28,29]. Some authors have reported good results with sensors based on metamaterial structures and split ring resonators (SRR) combined with microwave techniques. Due to their extraordinary electromagnetic properties, metamaterial particles have been proposed in energy harvesting applications, filters, high gain or miniaturized antennas and sensors [29,30,31,32,33,34]. In [35] the design and development of a planar aligned gap and centered gap rectangular multiple split ring resonator to measure dielectric permittivity from 1 to 10 with a maximum sensitivity of 0.032/Δε is presented. A different alternative has been explored in [36] with the use of wireless sensing system based on the implementation of two types of substrate-integrated-waveguide (SIW) for dielectric permittivity measurement in liquids, which operates up to 4 GHz and reported a sensitivity of 1.26 MHz/Δε. Metamaterial resonators have also been used in biosensing applications, where single rectangular or circular resonators in combination with transmission lines [11,37] or an array of resonator [38] are used for DNA sensing or label-free stress biomarkers. Some interesting works have been reported recently, for example a non-invasive microwave method based in squared-shaped complementary split-ring resonator (CSRR) is presented in [39]. This CSRR is used to measure the thickness and permittivity of multilayer electrical structures. Changes in resonance frequency depend on the thickness and permittivity of the multilayer dielectric sample below the ground plane (CSRR had been etched on the ground plane of a microstrip line). The analysis of sensor’s size optimization improved the resolution in permittivity and thickness measurement. On the other hand, a novel structure with two- and three-layer magnetic coupled SRRs small resonators, have been proposed in [24] to measure the permittivity of SUTs. Compared with the two-layer resonator, the proposed three-layer resonator has higher sensitivity, better stability and stronger anti-jamming ability from the external interference. The obtained resonance frequency shift was used to feed an algorithm in the post-processing stage, and based on the results, a formula that relates the dielectric constants to the resonant frequencies was proposed. The use of an algorithm gives an advantage in the measurement, especially when dealing with noise and unavoidable test errors. In other work, a promising alternative for analyzing biological samples based on the combination of SRRs and microfluidic channels filled with smaller volumes has been employed [40,41,42,43,44]. A clear example of this methodology was proposed in [38], where a compact microwave resonator capable of performing characterization of the complex permittivity of fluids was proposed. The developed sensor is based on a quarter-wavelength resonator designed on coplanar waveguide. It employs a change in the resonance frequency for dielectric characterization. The sensor presents a high sensitivity, but the measurement accuracy for small changes in permittivity is affected. In a similar way, a sensor based on CSRR is presented in [39]. In this case, the sensor is used to provide a larger area of fringing electric field that increases the effective interaction area with the sample. One microfluidic channel attached to the CSRR delivers the fluidic sample to the sensing area to determine the complex permittivity of liquids based on changes in the resonance frequency. Likewise, a different approach proposed a CSRR-loaded patch sensor as a microfluidic ethanol chemical sensor. It has a microfluidic channel integrated on the most sensitive area of the CSRR slot [40].

An important achievement in relation to the robustness of microwave sensors against environmental factors is presented in [43], where a microfluidic sensor for dielectric characterization of liquids in real time is presented. The sensor is composed of a microstrip SRR-loaded splitter/combiner configuration etched on a substrate, and two microfluidic channels placed on top of the gap region of the SRRs. The sensor works in differential mode, and the sensing mechanism is based on frequency splitting. If the axial symmetry is disrupted, two transmission zeros arise, and the difference in magnitude (notch depth) and frequency between such transmission zeros is indicative of the difference in the dielectric properties (complex dielectric constant). The advantages of differential mode are also presented in [45], where a microwave sensor based on a pair of symmetric uncoupled lines, each one loaded with an open complementary split ring resonator (OCSRR), has been proposed. The sensing principle is based on the measurement of the cross-mode insertion loss, very sensitive to small perturbations between the reference liquid and the liquid under test (each in a different channel). Similar to [43], in [46] the authors also show a differential microwave sensor based on a pair of uncoupled microstrip lines, each one loaded with a split ring resonator (SRR). The sensor is applied to the measurement of electrolyte concentration in deionized (DI) water. Compared to [43,45], this sensor uses a different principle, besides, a novel via-less SRR-based sensor with improved sensitivity is presented. Finally, an interesting alternative for developing sensors for industrial applications was introduced for first time in [20]. In that work the authors proposed a split-ring resonator (SRR)-based sensor for the detection of solid thickness and relative permittivity characterization of solid and liquid materials. The structure was composed of two SRRs hosted in a microstrip transmission line. A shift in frequency of the notch introduced by the resonators in the transmission coefficient is related to a change in the effective permittivity of the structure when the sensor is covered with any solid or liquid material. This work is very interesting because the proposed sensor is fully submersible and reusable. Submersible sensors offer a great alternative in industrial applications such as the measurement of some solvents and oils, because the amount of sample is large enough so a sensor can be directly submerged like a probe. It would help to implement this kind of sensor in an easier and more cost effective way than microfluidic based-sensors.

In this work, a microwave sensor based on an antenna-coupled split ring resonator with monopole insertions to measure the dielectric permittivity of liquid substances is presented. The sensor was designed to identify unknown dielectric permittivity of liquids in a wide range. This proposed sensor reaches a great sensibility, high Q-Factor and presents a good repeatability, which was predicted by simulation results and experimentally validated. The proposed alternative has some important advantages compared with previous works due to the fact that the sensor can be reusable, the measuring technique is not destructive, the sensor is submersible, and it allows real-time measurements of the variation of permittivity. To analyze the behavior of the sensor structure, finite element method (FEM) was used. In addition, the device was fabricated and its performance corroborated at detecting changes in dielectric permittivity of different liquid samples. The organization of the manuscript is as follows: the working principle, sensor design and characterization process are presented in Section 2; Section 3 shows experimental measurements and presents a discussion of the obtained results; finally, conclusions are presented in Section 4.

## 2. Materials and Methods

### 2.1. Theoretical Model

**Figure 1:**
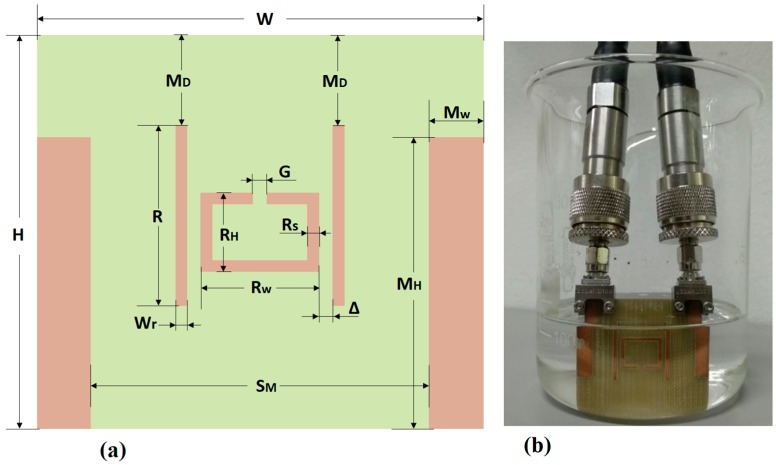

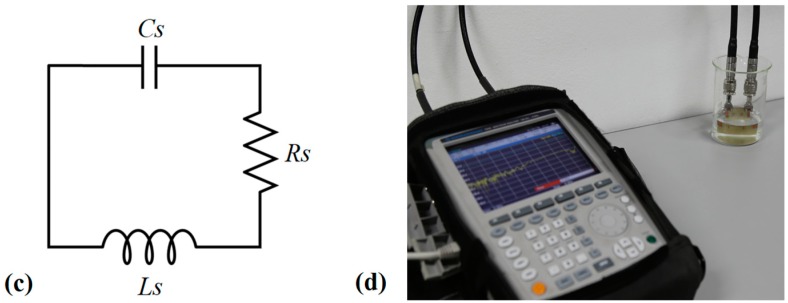
(**a**) Schematic of the proposed sensor device. (**b**) Picture of the sensor device immersed in the material under test (MUT). (**c**) Equivalent electrical circuit of the proposed SRR. (**d**) Sensor’s dipoles connected to the analyzer.

In this work, a rectangular split ring resonator (SRR) is placed between a group of printed monopole antennas on the same plane, as show in the Figure 1a. The SRR structure is based on a metal loop with a square shape and it is used as a transducer to detect dielectric permittivity changes in the surrounding medium. The monopole excitation is employed to send a magnetic field perpendicular to the ring surface, which induces a current through the rectangular SRR. In the past, similar configurations have explored this kind of excitation [47,48], however in those cases the monopoles are located externally, whereas in our case the monopoles are integrated in the same PCB in order to increase the stability, alignment, compactness and performance. The SRR can be modeled using an equivalent resonant LC circuit as previously demonstrated by Baena et al. [49]. The equivalent LC circuit is shown in Figure 1c and its resonant frequency can be obtained using Equation (1):

(1) $$f_{r}=\frac{1}{2\pi \sqrt{L_{s}C_{s}}},$$

where *L_s_* and C_s_ represent the self-inductance and the distributed capacitance due to the gap of the SRR respectively. However, it is important to keep in mind that the capacitance can be decompose into two capacitance terms as indicated in the equation (2) [42,50]. The first one is the capacitance without the sample which includes the capacitive effect due to the channel walls, the dielectric properties of the substrate, the surrounding space, etc. The second term, describes the contribution due to the variation of the dielectric permittivity of the surrounding medium [42], where the dielectric permittivity of the sample (ε_sample_) could be a complex parameter and its depends of the used sample:

(2) $$C_{s}=C_{0}+\varepsilon_{\mathrm{sample}}C_{c}$$

From the above, a change in the relative permittivity of the surrounding medium results in a shift of the resonant frequency. Therefore, this principle can be exploited to measure variations in liquids, such as alcohols, oils and biological samples.

### 2.2. Sensor Design

Figure 1a shows a 2D schematic of the proposed sensor device, which consists of a rectangular SRR placed between a pair of monopole antennas used for exciting the resonator, of 28 mm of height (M_H_) and 1.5 mm of width (M_W_). In addition, a pair of parasitic elements with rectangular shape whose height (R) is 16 mm and width (W_R_) is 1 mm, are inserted to improve the signal transmission. Each one of these rectangular elements are positioned at 1.25 mm from the resonator. The backside of the board is completely covered with copper (ground), and the overall area of the proposed sensor is 1750 mm mm^2^ (35 × 50 mm) which makes it look as a compact device compared to previous dielectric sensors [24,25,26,51]. Other important parameters related with the proposed structure are summarized in Table 1. To manufacture the prototype of the proposed sensor, a CNC machine for printed circuit boards (LPKF ProtoMat D104, LPKF Laser & Electronics AG, Hanover, Germany) was used. The sensor is built on commercial dielectric substrate FR4, which have a relative permittivity (ε_r_) of 4.4, loss tangent (tan δ) of 0.019, a thickness of 1.6 mm and a copper layer of 35 μm. The ends of each monopole are soldered to 50-Ω SMA male connectors (SOUTHWEST 292-07A-5, Southwest Microwave, Tempe, AZ, USA) for testing purposes, as illustrated in Figure 1c.

### 2.3. Characterization of Sensor

A FSH8 vector network analyzer (VNA, Rhode & Schwartz, Munich, Germany) with two ports is used in order to validate the performance of the proposed sensor. The procedure used to perform the measurements, as shown in Figure 1d, consisted of submerging the whole sensor in 100 ml of the material under test (MUT), to guarantee total interaction between the sensor and the MUT (as seen before, this is a non-destructive procedure). During the test, the response of the S_21_ parameter is monitored in real time with the VNA from 4 GHz to 5.4 GHz in order to detect variations in the surrounding medium caused by alterations in the resonant frequency of the proposed sensor. This procedure is repeated *N* times for each material, using *M* materials as MUT.

In order to obtain the actual dielectric permittivity of every sample measured in this work, the 85070E Dielectric Probe Kit (Agilent, Santa Clara, CA, USA) is used at room temperature and at a humidity level within the 65 RH% ± 5 range. From this experiment, the determined dielectric permittivity of the employed samples are 20.7, 21.8, 33.1 and 37 for acetone at 96% purity, propyl alcohol at 92% purity, methanol at 92% purity, and ethylene glycol at 93% purity, respectively (Figure S1).

## 3. Results and Discussion

**Figure 2:**
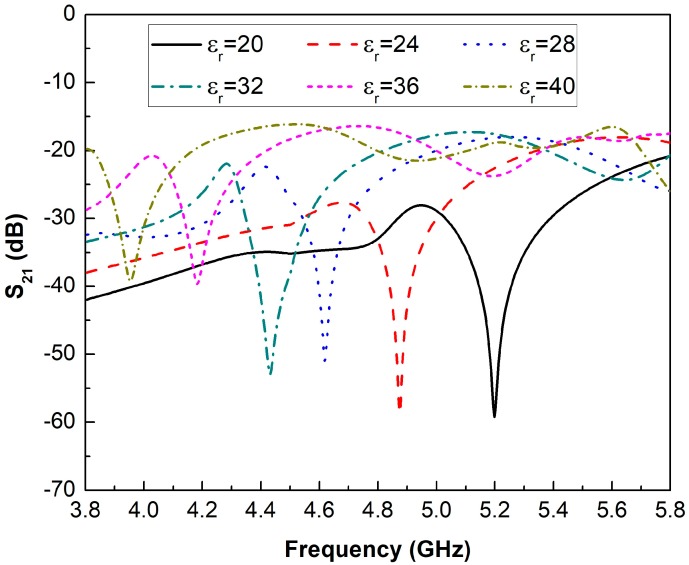
Simulated results of the transmission response (S_21_) when the dielectric permittivity is changed from 20 to 40.

The proposed sensor is designed and optimized using the full wave electromagnetic solver ANSYS HFSS. Figure 2 shows the analysis of the S21 parameter when the dielectric permittivity of the surrounding medium is changed from 20 to 40. This plot shows that transmitted electromagnetic energy becomes minimum at the resonance frequency of the designed structure, as the maximum energy couples to the resonator at the resonance frequency. Similarly, the resonance frequency of the resonator shifts towards lower frequencies as the permittivity of the surrounding dielectric medium of the MUT increases.

**Figure 3:**
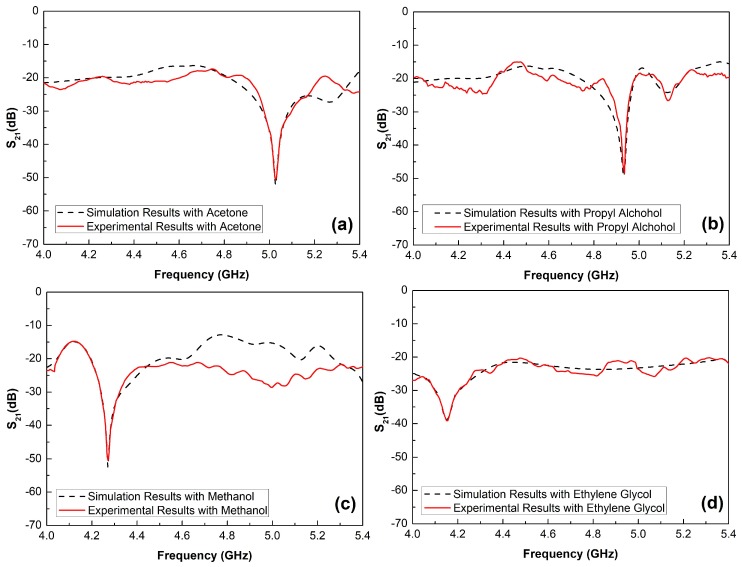
Simulated and experimental results obtained for (**a**) Acetone (ε = 20.7), (**b**) propyl alcohol (ε = 21.8), (**c**) methanol (ε = 33.1) and (**d**) ethylene glycol (ε = 37).

The previous model, presented in the Section 2.1, predicted this result. When the dielectric permittivity of the surrounding medium in which the sensor is immersed increases, the second term of Equation (2) increases too, and the frequency decreases in Equation (1). In this work, the changes in the resonance frequency of the sensor are exclusively due to changes in the surrounding medium, since it does not present changes in the geometrical parameters due to temperature or strain variations. Thus, when the dielectric permittivity changes from 20 to 40, the resonance frequency is lowered from 5.198 GHz to 3.955 GHz. In addition, the quality factor of the proposed structure decreases for higher values of dielectric permittivity. For example, when the sensor device is submerged in a sample whose dielectric permittivity is 20, a quality factor close to 206.83 is obtained, while this parameter decreases to a relatively high value of 86.28 when the dielectric permittivity of the surrounding medium is equal to 40. Further, the behavior of the designed sensor is validated. For this step, four different samples of liquids with dielectric permittivity from 20 to 40 are employed. Figure 3 shows a comparison of the simulated (black line) and the experimental (red line) results in a wideband frequency response from 4 GHz to 5.4 GHz. As mentioned in Section 2.3, two port measurements are taken with the Rhode & Schwartz FSH8 VNA with the sensor submerged into the MUT as is depicted in Figure 1d. To carry out these measurements, acetone, propyl alcohol, methanol and ethylene glycol are used due to the fact that their dielectric permittivity values are into the selected operating range. As seen, the experimental results have a great agreement with the simulated ones observed, which validates the design of the fabricated prototype. The small differences between them can be due to small fabrication tolerances in the engraved process with the CNC machine. However, the slight frequency shifts between the simulated and measured results does not affect the characterization of materials with the proposed sensor.

**Figure 4:**
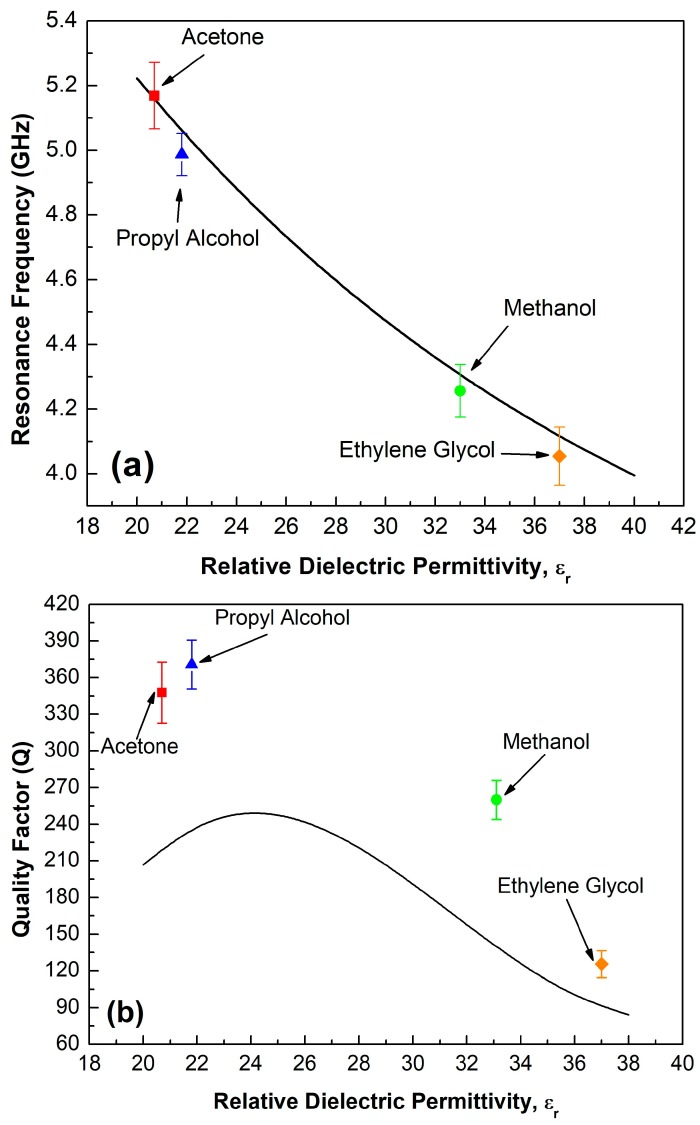
(**a**) Resonance frequency in function of the dielectric permittivity of different liquids. (**b**) Quality factor analysis of the proposed sensor within the operating range. The point in the plot are the mean value of the resonance frequency and the bars are representative of the standard deviation.

In order to evaluate the repeatability of the sensor, each experimental measurement was repeated 10 times inside a temperature-controlled room at 22 ± 2 °C and humidity within the 65 RH% ± 5 range. Next, the data of each measurement was processed to obtain the resonance frequency and its mean value with the respective standard deviation. Results are illustrated in Figure 4a. The obtained standard deviation values are 0.102, 0.065, 0.081 and 0.090 GHz for the tested materials acetone, propyl alcohol, methanol, and ethylene glycol, respectively. These results indicate that the sensor shows high repeatability due to a very small value of standard deviation for the measured results. Besides, the behavior of this sensor shows clearly that, the resonance frequency shifted down according to the increasing value of relative dielectric permittivity of the liquid and this presents a trend with decreasing exponential fitting, which is expressed as follows:

(3) $$f_{r}\left(GHz\right)=5.0855e^{\left(-\frac{\varepsilon }{22.30237}\right)}+3.1488$$

Now, from the above curve it is possible to characterize any material with relative dielectric permittivity within the range from 20 to 40.

Therefore, this device can be employed to determine the dielectric constant of unknown MUTs through the retrieve method. For example, the proposed sensor can be employed to measure the variations of the alcohol concentration when it is mixed with water or other solvent. As demonstrated in this work, the proposed sensor could be used in industrial applications where the alcohol concentration is an important parameter. In the same way, the proposed structure presents good performance when it is compared with other works [20,24,36,50,52] because, as it is clear from the Equation (3), a displacement of 1.2282 GHz when the dielectric permittivity of the sample is varied from 20 to 40 (i.e., 23.52% relative frequency shift) is easily obtained. It is comparable, for example, with the results reported by Xu et al. [24] with a relative frequency shift of 24.24% for a microwave sensor that allows to characterize samples with dielectric permittivity values ranging from 1 to 10. Moreover, the submersible printed sensor proposed by Galindo-Romera et al. [20] reported a relative frequency shift of 22.04% for a configuration that it is implemented in the characterization of liquid with dielectric permittivity between 2.45 and 22.52.

Finally, the quality factor of the proposed structured was evaluated in each case. Figure 4b shows a comparison between the obtained theoretical and experimental results. The theoretical model shows that a maximum quality factor of 252 is obtained when the dielectric permittivity of the surrounding medium is close to 24 and it decreases strongly to 84 by samples whose is near to 38 as was predicted by the theoretical model. The experimental results show the same trend observed in the theoretical model. The experimental results show a same trend that the theoretical model, which validate the response of this sensor. However, as it is evident from the results illustrated in Figure 3, the Full Width at Half Maximum (FHWM) in experimental results is lower than theoretical results, for this reason, we obtain a higher quality factor with the experimental data. A mean quality factor of 347.677, 370.834, 259.967 and 125.438 were obtain experimentally for acetone, propyl alcohol, methanol and ethylene glycol respectively. Thus, the equation that describe the behavior of the Q-factor of the proposed sensor is can be expressed as:

(4) $$Q=-2565.78299+295.39906\varepsilon_{r}-9.94942\varepsilon_{r}^{2}+0.10555\varepsilon_{r}^{3}$$

From the obtained results, a completely passive, integrated, simple design, low cost and compact sensor device has been proposed and demonstrated. Likewise, the proposed sensor shows a high sensitivity and good quality factor when it is employed in the dielectric characterization of liquid samples like alcohols, which is desirable in industrial environments. The proposed structure presents several advantages in comparison with other previous alternatives. For example, it is a non-destructive technique because the sensor can be used like a submersible probe. On the other hand, this device allows obtaining high repeatability in the measurements in order to give reliable data to the users, and finally this one can be implemented in a wide range of applications because it is demonstrated it can be used to characterize dielectric materials with relative dielectric permittivity from 20 to 40.

## 4. Conclusions

A non-invasive, submersible and reusable material permittivity sensor device, based on monopole excited split ring resonator is proposed, validated and analyzed in this work. The operating principle of the sensor is based on the measurement of the resonance frequency shift as a function of the relative permittivity, which is used to detect changes in a wide range. Thus, the proposed sensor could be employed to obtain the permittivity of unknown liquids. The sensor’s structure was simulated at introducing it in several samples with different dielectric properties each one. After adjusting the sensor’s design, a prototype was obtained, and its performance was experimentally validated using several types of liquid samples as a material under test (MUT). The simulated and measured results evidently shown that the proposed sensor provide competitive sensibility, great repeatability, compact size and high Q-factor when it is employed to characterize unknown dielectric materials whose dielectric permittivity is within the range from 20 to 40. As a matter of fact, it was experimentally obtained a good matching between simulations results and measurements. In addition, the device has demonstrated an excellent performance, simple design and economic manufacturing process, which becomes this sensor device an attractive candidate for a fully integrated platform dedicated to many industrial applications, mainly liquid samples characterization in a wide range.

As a future improvement, it would be interesting to develop a version of the proposed sensor that allows characterizing the dielectric permittivity in multiple-bands simultaneously. It could be interesting because—in general—the dielectric permittivity of materials has a strong dependence with the frequency.

## Figures and Tables

**Table 1:** Dimensions of the proposed sensor based on a monopole-coupled SRR.

Variable	Dimension (mm)
Substrate width (W)	40.0
Substrate height (H)	35.0
Monopole width (M_w_)	1.5
Monopole height (M_H_)	28.0
Rectangle height (R)	16.0
Resonator width (R_W_)	10.5
Rectangle width (W_R_)	1.0
Resonator height (R_H_)	7.0
Resonator separation (R_s_)	1.0
Gap (G)	1.5
Monopoles separation (S_M_)	24.5
Separation between resonator and monopole (Δ)	1.25
Monopole distance (M_D_)	8

## Supplementary Materials

The following are available online at https://www.mdpi.com/1424-8220/19/8/1936/s1, Figure S1: Experimental results obtained with the Agilent 85070E Dielectric Probe Kit for (**a**) acetone at 96% purity. (**b**) Propyl alcohol at 92% purity. (**c**) Methanol at 92% purity. (**d**) Ethylene glycol at 93% purity.
