# Supplementary material for: A Submersible Printed Sensor Based on a Monopole-Coupled Split Ring Resonator for Permittivity Characterization
Source: Sensors (Basel). 2019 Apr 25;19(8):1936. doi: 10.3390/s19081936 (PMC6515438; doi:10.3390/s19081936)
Supplement: Supplementary file 1 [file sensors-19-01936-s001.pdf]

Supplementary Material: A Submersible Printed Sensor Based on a Monopole-Coupled Split Ring Resonator for Permittivity Characterization

Erick Reyes-Vera [1,*], G. Acevedo-Osorio [1], Mauricio Arias-Correa [2] and David E. Senior [3]

[1] Department of Electronic and Telecommunications Engineering, Instituto Tecnológico Metropolitano, Medellín 050012, Colombia; gabrielacevedo84945@correo.itm.edu.co

[2] Department of Mechatronics and Electromechanical Engineering, Instituto Tecnológico Metropolitano, Medellín 050034, Colombia; mauricioarias@itm.edu.co

[3] Department of Electrical and Electronic Engineering, Universidad Tecnologica de Bolivar, Cartagena 130001, Colombia; david.senior@ttm.com

* Correspondence: erickreyes@itm.edu.co; Tel.: +57-4-4600727

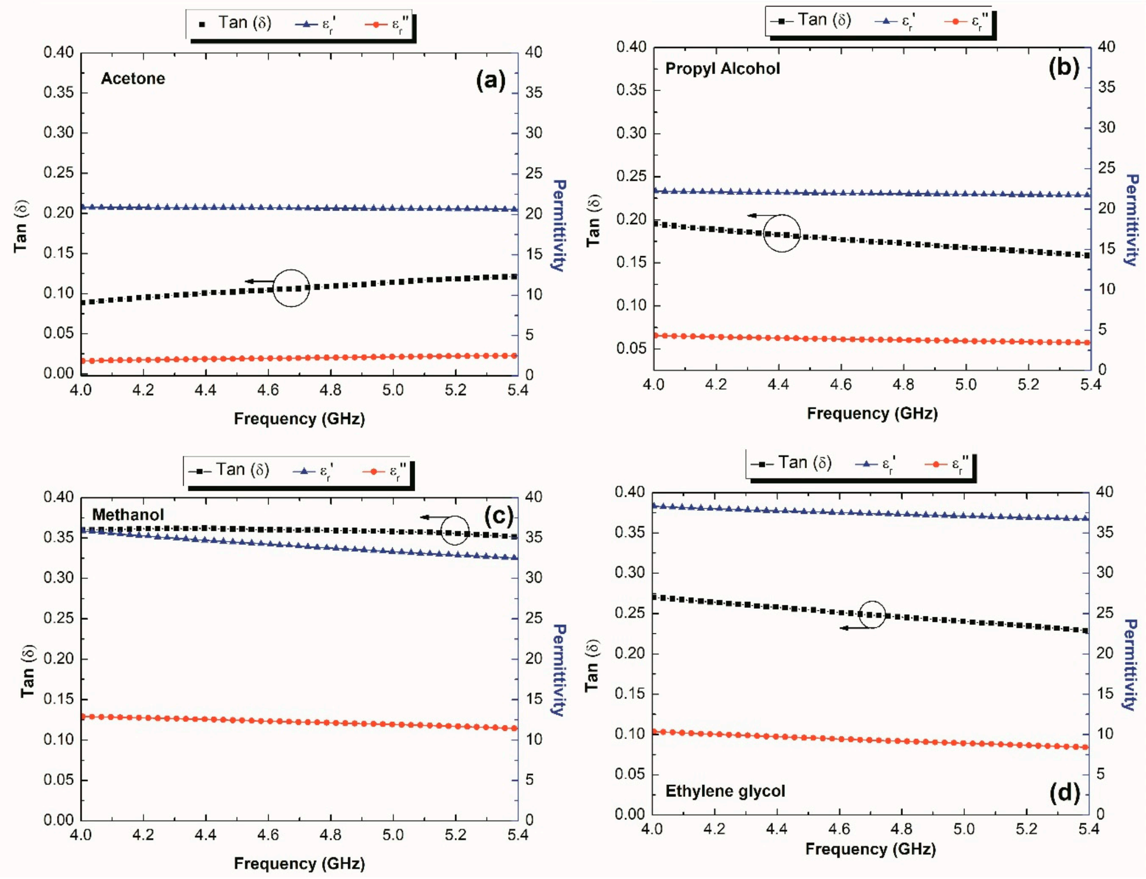

Figure S1. Experimental results obtained with the Agilent 85070E Dielectric Probe Kit for (**a**) acetone at 96% purity. (**b**) Propyl alcohol at 92% purity. (**c**) Methanol at 92% purity. (**d**) Ethylene glycol at 93% purity.

In the proposed work, we use the Agilent 85070E Dielectric Probe Kit to obtain the dielectric permittivity (complex) of the four samples used in this work. It was employed in order to validate the characteristic curve of the proposed sensor. As mentioned in the manuscript, we use acetone at 96% purity, propyl alcohol at 92% purity, methanol at 92% purity, and ethylene glycol at 93% purity. Then, in this section, we report the experimental results obtained with the kit when the probe was

immersed in 1000 ml of the sample. As it is evident from the four Figures, we report the real part of the permittivity (ε_r'), the imaginary part of the permittivity (ε_r'') and the loss tangent.
